# A Rare Case of a Right Atrial Paraganglioma in an Individual with the SHDB Mutation

**DOI:** 10.1155/2022/1140976

**Published:** 2022-10-03

**Authors:** Ian Lancaster, Carlos Nunez, Andrew Willinger, Christiano Caldeira, Jeffrey Aufman

**Affiliations:** HCA Healthcare/USF Morsani College of Medicine GME/Largo Medical Center, 201 14th St. SW, Largo, FL 33770, USA

## Abstract

Paragangliomas are extra-adrenal chromaffin cell tumors. A small percentage of these tumors can be found in the thoracic cavity and, when in the heart, are typically in the left atrium. In this case report, we discuss the case of an individual with a history of several paragangliomas with the *SHDB* mutation who was found to have two cardiac paragangliomas in the right atrium.

## 1. Introduction

Paragangliomas are extra-adrenal chromaffin cell tumors [1]. The approximate incidence of paragangliomas and pheochromocytomas is approximately 6-7 cases per million individuals per year [2]. The incidence of pheochromocytoma and paragangliomas increases through adulthood until age 60–79 years old, where in the incidence is 8.85 and 14.68 cases per 100,000 people annually for males and females, respectively [2]. In addition, approximately 1.2% of paragangliomas occur in the thoracic cavity [2]. Also, approximately 1% of all paragangliomas are cardiac paragangliomas [2].

There are two general classes of cardiac paragangliomas, sympathetic and parasympathetic. Sympathetic paragangliomas produce catecholamines (particularly norepinephrine or epinephrine) while parasympathetic paragangliomas are usually nonfunctional and mainly located in the head and neck [3]. Cardiac paragangliomas arise from the visceral or branchiomeric autonomic paraganglia resulting in the left atrial and aortic body tumors, respectively [3]. Although cardiac paragangliomas are commonly described in all cardiac chambers, left atrial paragangliomas are the most common, followed by aortic body tumors [3]. Mutations in the succinate dehydrogenase complex unit B (*SDHB*) or succinate dehydrogenase complex subunit D (*SDHD*) genes are common in individuals with thoracic paragangliomas [4].

Frequently, cardiac paragangliomas are close to the great vessels and can involve any of the cardiac cavities [3, 5]. Moreover, these tumors infiltrate cardiac tissues or the pericardial space, which can result in features of cardiac tamponade and make resection challenging [5–7]. In instances when the atrioventricular groove or extracardiac structures are involved, resection may become impossible [5]. Medical management prior to resection of these tumors is aimed at preventing the perioperative complications including hypertensive crisis, cardiac arrhythmias, or even myocardial infarction with the use of alpha- (*α*-) adrenoceptor and beta- (*β*-) adrenoceptor blockers at least 2 weeks prior to the surgery [8]. In this case report, we discuss the case of a 26-year-old male whom presented for surgical management of his right atrial paraganglioma.

## 2. Case Presentation

A 26-year-old male with a past medical history of pheochromocytoma and multiple head and neck paragangliomas presented for worsening shortness of breath for the last “couple of weeks.” His dyspnea was present both on exertion and at rest. He had no associated chest pain, palpitations, sweating, nausea, or vomiting. Additionally, he states that he had no similar events to this shortness of breath. He had no reported “significant” change in weight over the last 3 months and states that his hypertension had been “well managed” with his reported systolic blood pressures ranging from 110 to 130 on his current antihypertensive regimen. At an outside facility, an echocardiogram and cardiac magnetic resonance imaging were performed and a right atrial mass was appreciated prompting transfer to a specialized cardiac care center. He did not require supplemental oxygen; his lungs were clear to auscultation bilaterally, and no murmurs were appreciated on physical exam. He was admitted for a planned resection of his cardiac tumor with review of preoperative imaging to be completed for assistance in guiding the operative approach.

As previously stated, his past medical history was significant for multiple paragangliomas in his head and neck, pheochromocytoma requiring adrenalectomy, and an *SDHB* gene mutation. His first case of an identified paraganglioma occurred when he was 14 years of age and was found to have the *SDHB* mutation. Per report, he had undergone at least 6 surgical excisions for a variety of paragangliomas though the dates of these procedures were unknown. His most recent surgical excision was 7 years prior to presentation per patient and family report. In addition, his past medical history also includes adrenal insufficiency following adrenalectomy, hypertension, asthma, and tobacco use. His family history was significant for pheochromocytoma in his sister, father, uncle, and paternal grandfather with each requiring surgical excision and no further recurrences of catecholamine secreting tumors in those family members. Additionally, his mother had a history of hypertension.

His medication regimen as an outpatient included hydrocortisone for adrenal insufficiency as well as carvedilol, amlodipine, and doxazosin for management of his hypertension associated with his paragangliomas.

Lab work following transfer was performed and demonstrated a hemoglobin of 13.9 g/dL, white blood cell count of 7.0 × 10^9^/L, platelet count of 325 × 10^9^/L, sodium of 142 mEq/L, potassium of 3.5 mEq/L, creatinine of 0.91 mg/dL, and blood urea nitrogen (BUN) of 9 mg/dL. His coagulation studies were unremarkable, and liver function studies did not identify any abnormalities. His serum troponin was assessed preoperatively and was less than 0.04 ng/mL. His plasma norepinephrine level was 1186 pg/mL, and plasma normetanephrine level was 283.3 pg/mL. Additionally, his epinephrine level was less than 15 pg/mL and plasma metanephrine was 12.4 pg/mL. His baseline catecholamine levels as an outpatient were not available for review. Radiograph of the chest did not identify an acute cardiopulmonary disease.

**Figure 1:**
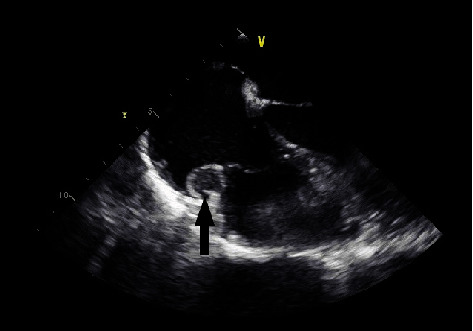
TEE horizontal view: right atrial mass (black arrow) in the area adjacent to the tricuspid valve. No visual obstruction of flow was identified on Doppler.

**Figure 2:**
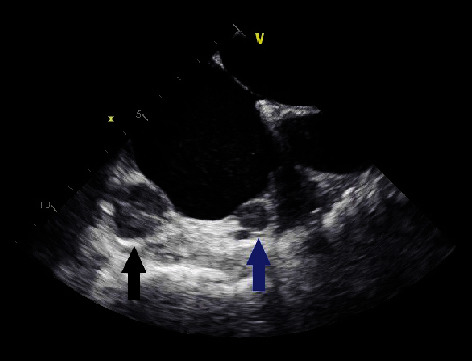
TEE horizontal view: right atrial masses in the area of the tricuspid valve and atrioventricular groove (blue arrow) and in the area of the atrioventricular groove (black arrow).

For surgical evaluation, review of prior imaging was performed. A transesophageal echocardiogram (TEE) identified two tumors within the right atrium (Figures 1 and 2). The presence of the cardiac tumors was then confirmed with cardiac magnetic resonance imaging.

As an outpatient, the patient had been compliant with his regime of carvedilol, amlodipine, and doxazosin. For preoperative management, he was continued on his outpatient medication regimen.

Following review of the echocardiogram and cardiac magnetic resonance imaging, surgical excision of the two masses were performed. The first tumor was small, measuring 1.0 cm in greatest dimension on the right atrium and close to the atrial appendage on the atrioventricular groove. The second tumor was larger and measured to be 2.5 cm in greatest dimension and attached to the atrioventricular groove, close to the right coronary artery (RCA) on the acute angle of the heart. For excision of the tumors, the patient was placed on full cardiopulmonary bypass. The tumors were then successfully excised and hemostasis was achieved using electrocautery, followed by the placement of several sutures. He had 500 cc of reported fluid loss and did not require transfusion of blood products.

**Figure 3:**
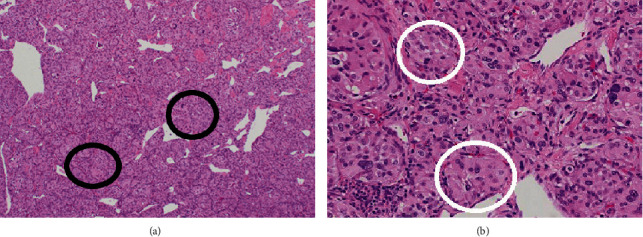
(a) Cardiac paraganglioma histology. Multiple large groups of characteristic Zellballen morphology (in black circles) with nests of uniform, polygonal chromaffin/chief cells surrounded by sustentacular support cells and well-formed vascular cuffing. (b) Cardiac paraganglioma histology. Characteristic Zellballen morphology (white circles) with nests of uniform, polygonal chromaffin/chief cells surrounded by sustentacular support cells and well-formed vascular cuffing.

**Figure 4:**
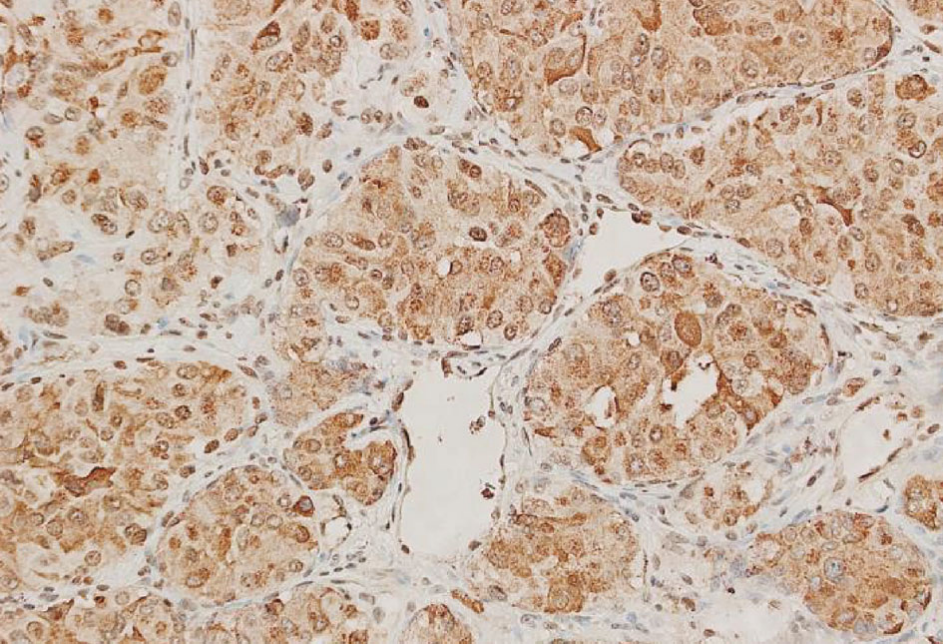
Cardiac paraganglioma histology. Chromaffin cells positive with chromogranin-positive immunohistochemistry stain.

After resection of the tumors, each excised mass was then sent to the pathologist for review. Histologic review confirmed paraganglioma for each of the resected tumors. Immunohistochemistry confirmed neoplastic cells which stained positive for chromogranin and GATA binding protein 3 (GATA-3) and a solid nest-like arrangement partially demarcated by S100-positive sustentacular cells in a “Zellballen” pattern (Figures 3(a), 3(b) and 4).

## 3. Discussion

Pheochromocytomas are chromaffin cell tumors located within the adrenal medulla while paragangliomas are extra-adrenal. Approximately 1.2% of paragangliomas are located within the thoracic cavity, most in the posterior mediastinum [1] and a minority of these tumors are located within the heart [3, 9]. In a prior review of cardiac paragangliomas, it was identified that 85% arise from the epicardium (including interatrial groove and atrioventricular groove) as well as the root of the great vessels (including aorta, pulmonary artery, pulmonary vein, and vena cava) [5]. Typically, these tumors are found in the left atrium, 41.9%, but have been found in all four chambers of the heart and rise to the branchiomeric and visceral autonomic paraganglia [3, 5, 9]. This patient's paragangliomas were found within the right atrium and, to our knowledge, only 6 cases have been reported within the right atrium [5]. Cardiac paragangliomas are highly vascular and derive their blood supply from the coronary circulation [5]. These tumors usually do to not invade to surrounding structures, but their adherence to the surrounding tissues is thought to be due to compression [5]. Upon histologic evaluation, paragangliomas are composed of “chief cells” grouped together in cell clusters referred to as a “Zellballen” surrounded by a capillary network [10].

**Figure 5:**
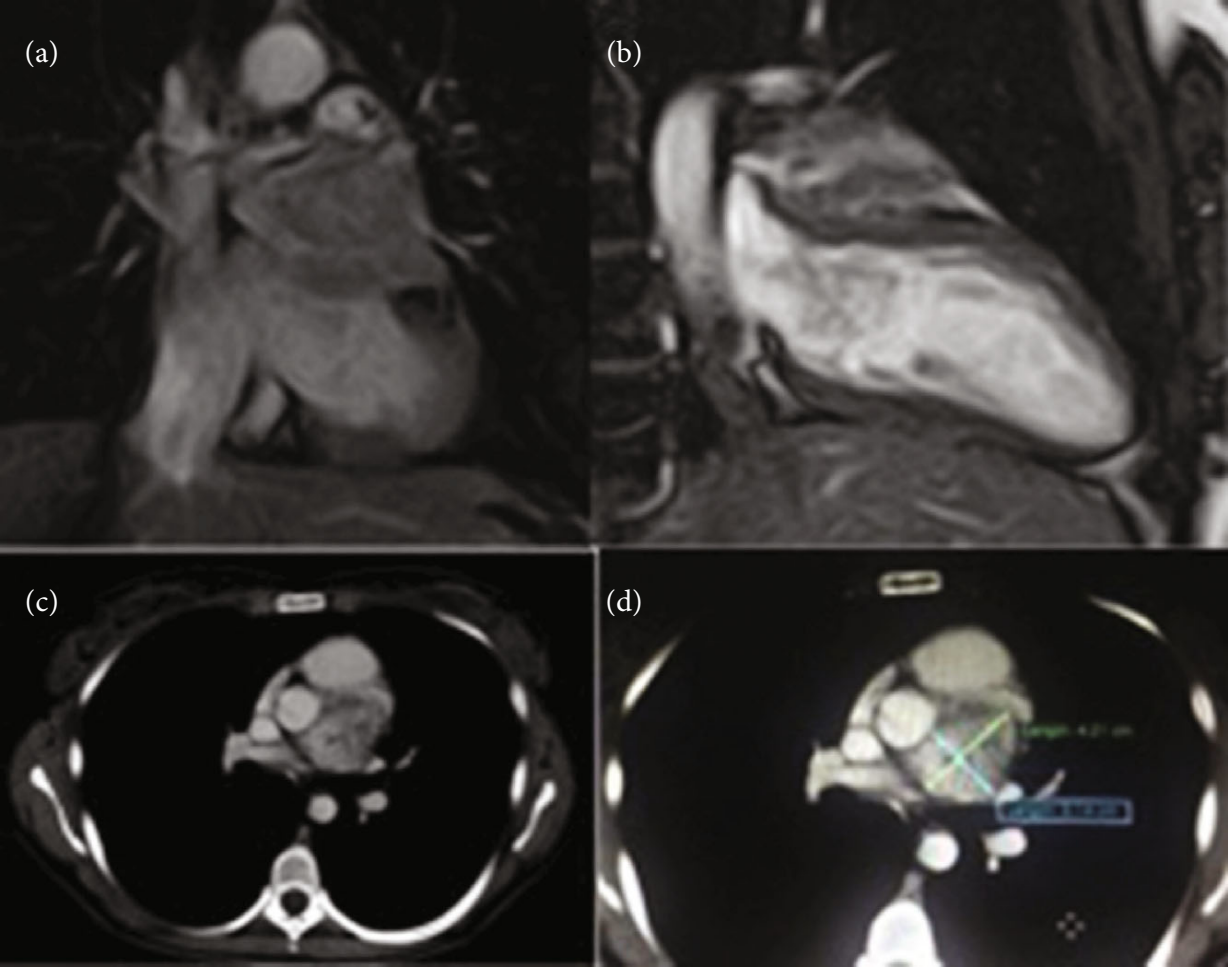
An example of Cardiac MRI with a cardiac paraganglioma [10]. (a) Coronal image with mass (isointense) in the area of the left atrium. (b) Two camber longitudinal view with the mass (isointense) in the left atrioventricular groove. (c, d) Axial view demonstrating the mass in relation to the aortic root (c) and pulmonary artery (d), respectively.

Evaluation for paragangliomas begins with the patient history and physical exam. Usually, patients complain of episodic headache, diaphoresis, tachycardia, or hypertension; and less commonly can present with palpitations, panic attacks, dyspnea, or orthostatic hypotension [11]. Then biochemical evaluation is performed with 24-hour urine catecholamines and metanephrines or serum metanephrines, with no test being preferred over the other [8]. The elevation in catecholamines is required for the diagnosis [12], but the degree elevation does not predict the invasiveness of the paraganglioma or pheochromocytoma [12, 13]. This is because multiple medications such as antipsychotics, serotonin reuptake inhibitors, and tricyclic antidepressants may alter the serum levels of catecholamines. In this patient's case, he did not complain of new symptomatology other than his shortness of breath. As he was already undergoing treatment for paragangliomas, he did not require biochemical confirmation. Next, imaging for identification of new paragangliomas is performed with either computed tomography (CT) or magnetic resonance imaging (MRI) (Figure 5) [14, 15]. In the case of cardiac paragangliomas both transthoracic and transesophageal echocardiograms can be used for evaluation of the tumor [1, 14]. Additionally, coronary angiography can be considered to outline the vasculature of the tumor prior to surgery as well as the opportunity to embolize potential vascular supplies to the tumor. After the tumor has been identified, further imaging may be required to evaluate for metastatic disease. Previously the 123-I meta-iodobenzyl-guanidine (MIBG) imaging was used to look for metastatic disease [1]. Now, current evaluation methods for detecting metastatic disease include positron emission tomography (PET) with or with computed tomography (CT), somatostatin receptor imaging using indium^111^- or Tc^99m^-labeled octreotide or magnetic resonance imaging [5, 16]. This patient had his tumors identified on echocardiogram for evaluation of his dyspnea. These tumors were then confirmed on cardiac magnetic resonance imaging. His outpatient PET scans were not available for review during the hospitalization but were reportedly negative.

The hemodynamic effects and management of cardiac paragangliomas and are dependent on their size, location, and presence of obstruction associated with the tumor. Additionally, paragangliomas are associated most commonly with a catecholamine surge leading to labile elevations in blood pressure which precipitate cardiovascular collapse, pulmonary edema, and occasionally acute respiratory failure [17]. This may be induced by a variety of stimuli of mechanical stressors or by psychological stressors such as anxiety or severe pain [18]. Additionally, hypotension from circulatory collapse is most commonly due to left ventricular dysfunction leading to left ventricular outlet obstruction [19]. In rare instances, the circulatory collapse may due to beta-2 adrenoreceptor stimulation by epinephrine which results in peripheral vasodilation thus precipitating the hypotension [18].

The definitive treatment for cardiac paragangliomas is surgical excision [20]. The major consideration for the majority of patients is the timing of the procedure as well as the surgical approach. The preoperative and intraoperative management of patients with paragangliomas is focused on prevention of hypertensive crises and hypotensive episodes [13]. This is achieved with alpha-blockade followed by beta-blockade and occasional use of dihydropyridine calcium channel blockers for blood pressure control [10]. As an outpatient, the patient was already on metoprolol, doxazosin, and amlodipine. Following admission, intraoperative management with intravenous phenylephrine, esmolol, and nicardipine was undertaken [13]. Due to the heterogeneity of paraganglioma locations in the heart, a wide variety of surgical maneuvers can be employed to achieve surgical excision. Included are transection the inferior vena cava, superior vena cava, pulmonary artery, or aortic root replacement [11, 20]. In the analysis of 100 cardiac paragangliomas requiring surgical excision, approximately 10% had died due to extensive blood loss following surgical excision [4, 20]. In this patient's case, he was placed “off pump” prior to surgical excision and bypass was terminated only after electrocautery and suture placement had finished.

When individuals are considered poor candidates for surgical excision treatment options include chemotherapy and radionuclide therapy. Instances which may exclude patients from surgical excision include the location of the tumor, most typically head and neck paragangliomas; the extent of tumor invasion; and the size of the tumor, especially when greater than 5 cm in diameter [13, 16]. Multiple chemotherapeutic regimens have been developed for paragangliomas and include the CVD regimen of cyclophosphamide, vincristine, and darcarbazine; temozolomide monotherapy; radionuclide pharmacotherapy such as conventional or high-specific activity ^131^I-MIBG; and peptide receptor radionuclide therapy either alone or in combination with octreotide or low-dose capecitabine [21]. In those with rapidly progressive and metastatic disease, especially in individuals with *SDHB* mutations, combination chemotherapy with CVD followed by temozolomide monotherapy is a first-line option [22]. In the patients who have failed standard temozolomide monotherapy, long-term low-dose temozolomide (metronomic regimen) in combination with lanreotide may be attempted [23]. Alternatively, in patients with slowly progressive metastatic disease, systemic radiotherapy with ^131^I-MIBG or ^177^Lu/^90^Y-DOTA peptides should be considered [16]. With regard to metastatic paragangliomas, treatment will include both surgical excision as well as consideration for treatment with chemotherapy, radionuclide therapy, thermal ablation, or external radiation therapy [13].

Next, identification of primary versus metastatic paragangliomas and pheochromocytomas is challenge. Primary tumors may develop in any location where sympathetic or parasympathetic ganglia are located [24]. Due to the wide dispersion and anatomic locations of both parasympathetic and sympathetic ganglia, the identification of primary versus metastatic tumors is challenging [24]. Thus, World Health Organization (WHO) in 2017 had updated the classification system to define metastatic disease as only sites where normal chromaffin tissue is not present [24]. This was done with the intention of avoiding misclassification of multicentric primary tumors as metastases. In this patient's case, though it had not been confirmed at the time, his tumor most likely represented a primary tumor due to the parasympathetic and sympathetic tissues located within the heart. Lastly, histology is required for a more complete evaluation for presence of metastatic paragangliomas [25]. This has led to the development of scoring systems such as the Grading System for Adrenal Pheochromocytoma and Paraganglioma (GAPP) in an attempt to predict the likelihood of future metastatic disease of a tumor [25]. This scoring system utilizes the histologic pattern, cellularity, presence of necrosis, vascular invasion, Ki67 labeling, and type of catecholamine secreting tumor to predict the likelihood of metastatic behavior [25]. Unfortunately, no single risk stratification has been endorsed for widespread use [26].

The majority of paragangliomas occur sporadically, but up to 30-40% are familial and associated with a variety of genes [27]. For individuals with newly identified paragangliomas, screening for germline mutations is recommended. Currently, the Von Hippel Lindau tumor suppressor gene (VHL); *RET* protooncgene, neurofibromin 1 (NF1); succinate dehydrogenase genes *SDHD*, *SDHAF2*, *SDHC*, *SDHB*, *SDHA*; transmembrane protein 127 (*TMEM127*); and MYC-associated factor X (*MAX*) [13]. The thoracic paragangliomas, including cardiac paragangliomas, are associated mostly with mutations in *SDHB*, *SDHD*, or *VHL* [13]. In our patient, he was previously evaluated to have the *SDHB* mutation. This gene as well as the other succinate dehydrogenase subunits (A, B, C, and D) are proteins within the mitochondria and are involved in both the Krebs cycle and electron transport chain [28]. Patient with the *SDHB*, *SDHC*, and *SDHD* genes are associated with cardiac paragangliomas and the *SDHB* mutation being the having the strongest association with cardiac paragangliomas [28]. Further, the *SDHB* mutation has been associated with extra-adrenal paragangliomas and metastases and is transmitted in an autosomal dominant inheritance pattern [4].

Depending on the patient's genetic mutation that and clinical scenario identified will determine the patient's surveillance and recommended follow-up. For individuals with the *SDHA*, *SDHB*, or *SDHD* mutations, MRI imaging including the skull base and neck, thorax, retroperitoneum and pelvis are recommended [13]. Additionally, it recommended that patients obtain a baseline measurement of metanephrines which is to then be followed by annual metanephrine levels [13]. Following surgical removal of pheochromocytomas or paragangliomas, it recommended to repeat imaging annually for 3 years following excision. In body areas that have had no tumor removal, it is recommended to repeat imaging every 3 years [13]. It is recommended that this follow-up be done with additionally follow-up with an endocrinologist. In our patient, it is recommended that he undergo MRI imaging of his thorax annually given his surgical removal of 2 right atrial paragangliomas in additional to other regularly recommended outpatient monitoring imaging.

For all individuals with first-degree relatives of an individual whom has been diagnosed with a paraganglioma and who also had an identified mutation should be offered predictive (or cascade) testing in the context of genetic counseling [13], the testing of first degree relatives has a 50% likelihood of identifying carriers of a mutation, most of which are typically asymptomatic [13]. These asymptomatic mutation carriers should be offered similar clinical surveillance and management as individuals whom have the mutation and a history of paraganglioma [13, 29].

## 4. Conclusions

Cardiac paragangliomas are extra-adrenal chromaffin cell tumors which are typically located in the left atrium but can present within all four chambers of the heart as well as the aortic and pulmonic valves. These tumors typically present with episodic tachycardia, hypertension, diaphoresis, and headache. Evaluation of paragangliomas will initially start with biochemical evaluation with 24-hour urine metanephrines or plasma metanephrines followed by magnetic resonance imaging or computed tomography. Treatment of these tumors is complete resection, and preoperative management consists of alpha-blockade followed by beta-blockade with consideration for use of calcium channel blockers for hypertension. When available, genetic analysis is recommended for individuals with paragangliomas and the *SHDB* mutation is associated cardiac paragangliomas.

## Data Availability

As a case report, the patient's individual information is not available for review, in accordance with HIPPA compliance. Prior to development and submission of the manuscript, written informed consent as obtained from the patient.
